# Arbuscular Mycorrhizal Fungi as an Important Factor Enabling the Adaptation of *Anthyllis vulneraria* L. to Zn-Pb-Polluted Tailings

**DOI:** 10.3390/plants12112092

**Published:** 2023-05-24

**Authors:** Marzena Sujkowska-Rybkowska, Anna Lisek, Beata Sumorok, Edyta Derkowska, Magdalena Szymańska, Lidia Sas-Paszt

**Affiliations:** 1 Institute of Biology, Department of Botany, Warsaw University of Life Sciences—WULS, Nowoursynowska 159, 02-776 Warsaw, Poland; 2 The National Institute of Horticultural Research, Konstytucji 3 Maja 1/3, 96-100 Skierniewice, Polandedyta.derkowska@inhort.pl (E.D.);; 3 Division of Agricultural and Environmental Chemistry, Institute of Agriculture, Warsaw University of Life Sciences—WULS, Nowoursynowska 159, 02-776 Warsaw, Poland; magdalena_szymanska@sggw.edu.pl

**Keywords:** symbiosis, arbuscular mycorrhizae, heavy metals, *Anthyllis vulneraria*

## Abstract

The old Zn-Pb-contaminated (calamine) tailings in southern Poland are spontaneously colonized by metal-tolerant *Anthyllis vulneraria* L. (*Fabaceae*), which can form simultaneously symbiotic association with nitrogen-fixing rhizobia and phosphorus-acquiring arbuscular mycorrhizal fungi (AMF). So far, fungal colonization and the AMF diversity of calamine-inhabiting legumes have been poorly studied. Thus, we determined AMF spore density in the substratum and the mycorrhizal status of nodulated *A. vulneraria* plants occurring on calamine tailings (M) and on a reference non-metallicolous (NM) site. The results indicate the presence of the *Arum*-type of arbuscular mycorrhiza in the roots of both Anthyllis ecotypes. Despite the presence of AM fungi in M plant roots, the dark septate endophyte (DSE) fungi (hyphae and microsclerotia) were occasionally also detected. Metal ions were accumulated mainly in the nodules and intraradical fungal structures rather than thick plant cell walls. Mycorrhization parameters (frequency of mycorrhization and intensity of root cortex colonization) for M plants were markedly higher and differed in a statistically significant manner from the parameters for NM plants. Heavy metal excess had no negative effect on the number of AMF spores, the amounts of glomalin-related soil proteins and AMF species composition. Molecular identification of AMF using PCR-DGGE analysis based on the 18S rDNA ribosomal gene by nested-PCR with primers AM1/NS31 and NS31-GC/Glo1 revealed similar genera/species of AMF in the roots of both Anthyllis ecotypes: *Rhizophagus* sp., *R. fasciculatus*, and *R. iranicus*. The results of this work indicate the presence of unique fungal symbionts, which may enhance *A. vulneraria* tolerance to heavy metal stress and plant adaptation to extreme conditions on calamine tailings.

## 1. Introduction

Soil contamination with heavy metals is a serious problem in agricultural and industrial areas in many countries around the world [1,2]. Heavy metals are non-degradable and can persist in the soil for a prolonged period, which poses a long-term threat to the natural environment and human health [3]. Since the 19th century, the mining of metal ores in southern Poland has been a source of dumps of waste material highly contaminated with heavy metals and metalloids [4]. The over 100-year-old calamine tailings in Bolesław are heavily contaminated with Zn (up to 42,983 mg·kg^−1^) and Pb (up to 2298 mg·kg^−1^) [4,5]. Additionally, the polluted sites are characterized by nutrient deficiency (especially phosphorous), low organic matter concentrations, strong insolation, and poorly developed soil structure, which are factors that significantly limit or prevent plant growth [6]. The excess of heavy metals has negative impact not only on plants but also on the biodiversity of microbiota [7]. Moreover, excessive concentrations of metals affect the physiological and biochemical processes, thus changing the functionality and stability of ecosystems [8,9].

One of the ways to limit the harmful effects of toxic metals on plants is the use of native rhizobacteria and/or mycorrhiza, and this strategy is termed rhizoremediation [10]. The metal-tolerant microorganisms in the rhizosphere either accumulate, detoxify, or remove heavy metals and thereby prevent the uptake of toxic metals by plants [10]. In metal-polluted soils, the roots of plants are mainly colonized by arbuscular mycorrhizal fungi (AMF) and/or dark septate endophyte (DSE) fungi [11]. Although heavy metals are toxic to most fungi, the root-colonizing AMF and DSEs possess the ability to survive and accumulate heavy metals without lethal effects [12,13,14,15,16]. The DSEs are a diverse group of Ascomycetes fungi with darkly pigmented (melanin deposition) and septate hyphae with characteristic dark microsclerotia inside the host root [17,18]. Arbuscular mycorrhizal fungi, from the order Glomales, form symbiosis with two-thirds of land plants, providing mainly phosphorus in exchange for carbon even on heavily degraded soils [13,14,15,16]. In AMF symbiosis, the fungi colonize the plant root cortex and maintain an extensive network of extraradical hyphae in the soil to absorb water and nutrients. Inside the root, the AM fungi form vesicles, hyphae and develop specialized structures called arbuscules to facilitate nutrient exchange between the partners [13]. In addition to nutrient uptake, AMF and DSE fungi improve plant tolerance to biotic and abiotic stresses, such as drought, salinity, heavy metal toxicity, and organic pollutants [18,19,20,21]. The mechanisms of metal tolerance in fungi include increased efflux and/or reduction of metal uptake, metal immobilization, e.g., intracellular sequestration by metallothioneins and phytochelatins, and extracellular binding by the cell wall and extracellular substances, such as glomalin [12,20,21,22,23]. Glomalin is defined as a glomalin-related soil protein (GRSP) produced by the hyphae and spores of AMF. These proteins have a strong reinforcing effect on soil aggregation and metal-sorption, and are critical in the survival of AMF and host plants on metal-contaminated sites [22,23,24,25,26]. Under stressful conditions, AMF produce more GRSPs to bind metals, thus improving plant tolerance to stress [25,26,27]. Similarly, the DSE fungi enhance plant growth in metal-contaminated soils [17,18]. The DSE fungi have developed melanin-dependent and melanin-independent detoxification and osmoprotection mechanisms against heavy metals [20]. These endophytes facilitate metal accumulation in plant roots and reduce metal migration to the shoots [18,26]. Thus, the AMF and DSEs colonizing plant roots can facilitate host-plant growth and enhance plant tolerance to toxic metals [27,28].

The species of legumes (*Fabaceae*) occurring on calamine wastes are an example of a highly specialized calamine flora adopted to extensive deposition of metals such as Zn and Pb [5,6,29,30]. Zinc and lead excess in soils can result in various alterations in non-tolerant plants, including reduced growth, inhibited seed germination, disturbed plant water and nutritional relations, inhibited photosynthetic and respiratory rates, imbalanced mineral nutrition, and can also cause oxidative damage and cell death [31]. Plants can develop resistance to heavy metals either through “avoidance” (by limiting uptake of metals) or through “tolerance” (coping with high levels of metals in their tissues) [31]. Calamine tailings are inhabited by legumes such as *Anthyllis vulneraria* L., adapted to such extreme conditions. *A. vulneraria* is indicated as a metal-tolerant species with a high potential for phytoremediation of soils contaminated with heavy metals [32]. Legumes have been proposed as suitable species for the remediation of metal-polluted soils, mainly because of their ability to symbiosis with nitrogen-fixing bacteria (rhizobia) and phosphorus-providing AM fungi [33]. These symbiotic plants have relatively high nitrogen concentrations and high phosphorus demands [33]. Rhizobia and AMF may have synergistic effects on the adaptation of legumes to metal-polluted soils through metal stabilization and improvement in metal tolerance by the host [34,35,36]. Soils under long-term exposure to heavy metals contain large adapted microbes [37]. Our preliminary studies show that Anthyllis plants are symbiotically active on old calamine wastes, interacting with both, metal-tolerant rhizobia, e.g., *Rhizobium* and *Bradyrhizobium*, and arbuscular mycorrhizal fungi [29,38]. However, not much is known about AMF communities inhabiting the Anthyllis roots, and the mycorrhization status of legumes colonizing calamine wastes has been poorly explored [29,38]. One technique used to identify AMF is denaturing gradient gel electrophoresis (PCR-DGGE). The PCR-DGGE is a method that separates DNA fragments on sequence differences [39]. PCR-DGGE has been used to identify AMF or to assess fungal compositions in the soil environment [40,41,42,43,44,45]. The technique has also been useful as a quality assessment tool in the collection of beneficial AM fungi [40].

In this paper, we present the results of research on the mycorrhization status and identification of genera/species of arbuscular mycorrhizal fungi colonizing the nodulated roots of calamine *A. vulneraria* ecotype. The information obtained revealed apparently special AM fungi that can play an important role in the adaptation of Anthyllis plants to stress caused by a permanent excess of metals in the tailings heap substrate and can serve as a potential biotechnological tool for successful restoration of metal-contaminated sites.

## 2. Results

### 2.1. Macroelement Concentrations

The chemical analysis indicates that the fertility status of the study sites is very low (Table 1). Total nitrogen (N) in both analyzed sites was very low, ranging from 0.99 g kg^−1^ dry mass of soil for NM to 1.01 g kg^−1^ for M tailings. Both sites also showed very low phosphorus (P) content, from 0.022% for NM to 0.03% for M tailings. Both analyzed sites showed high calcium (Ca) content but low Mg concentration. The tailings substrate showed significantly higher K and Mg content compared to the NM reference site (Table 1). The nutrient content in the shoots was also analyzed. The results showed that there was no significant difference in total nitrogen content between calamine plants and non-metal treated plants (Table 1). However, P, K, Ca and Mg showed a significant increase in calamine (M) plants compared to control non-polluted (NM) plants (Table 1).

### 2.2. In Situ Detection of Pb^2+^ and Cd^2+^ Location in Roots

**Figure 1:**
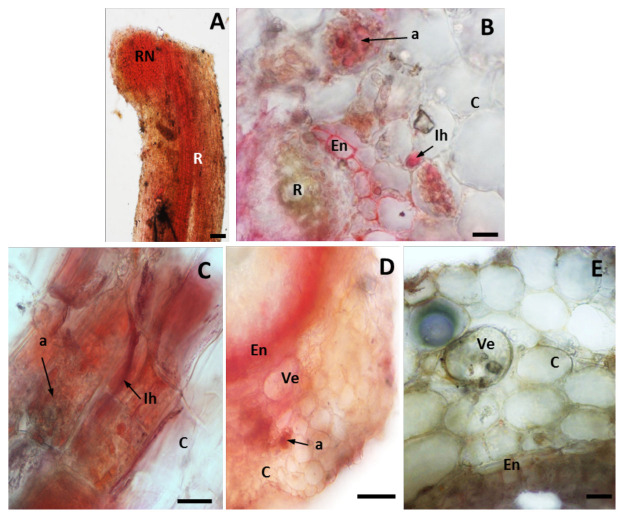
Heavy metal localization in the roots with nodules of the calamine (M) Anthyllis ecotype (**A**–**E**) or the control (NM) ecotype (F). (**A**) Visible strong metal-dithizone staining in the nodules (RN) and mycorrhizal roots (R) of M plants. (**B**–**D**) Intensive red staining present in the walls of root endodermis, and in walls of hyphae and vesicles and around AMF intraradical structures (hyphae and arbuscules). (**E**) Root of non-metallicolous ecotype and no red colour is visible. a—arbuscule, C—cortex, En—endodermis, Ih—intraradical hyphae, Ve—vesicles. Scale bars = 100 µm.

The complex of dithizone with Cd and Pb ions is red and the intensity of staining corresponds to the accumulation of metal in cells and tissues [46]. Intensive metal-dithizone staining was observed only in M plants, in roots and nodules (Figure 1). We observed localisation of absorbed metals around vesicles and arbuscules, and inside intracellular hyphae and also in cell walls of root endodermis (Figure 1A–D). No staining of control roots was observed (Figure 1E).

### 2.3. Root (Ultra)Structural Analysis

**Figure 2:**
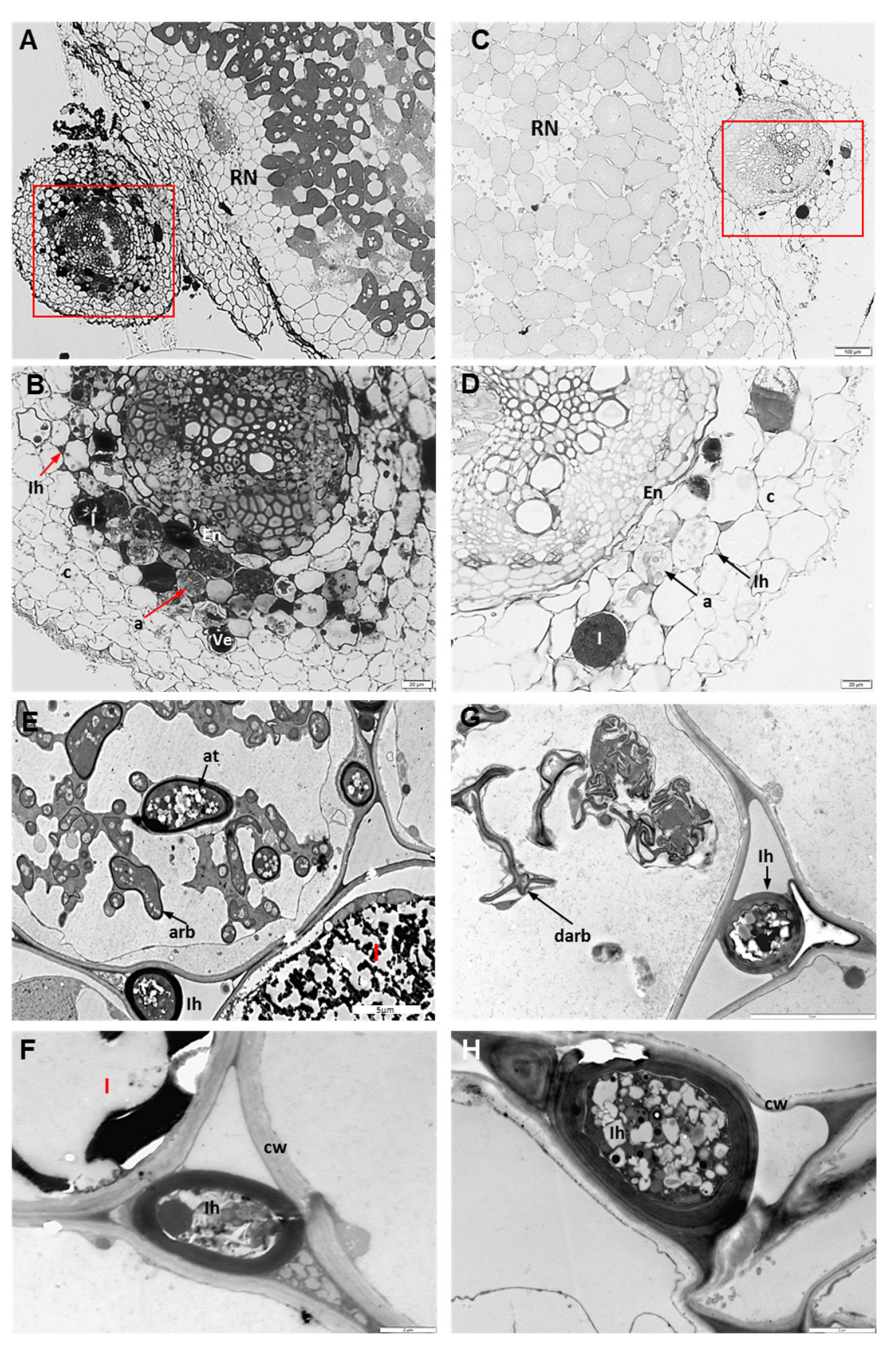
Transverse sections of mycorrhizal root nodules of Anthyllis plants from calamine tailings (M) (**A**,**B**,**E**,**F**) and reference site (NM) (**C**,**D**,**G**,**H**). Microscopic images of Anthyllis roots using LM (**A**–**D**) and TEM (**E**–**H**). (**A**,**C**) Red rectangles indicate the root fragment that is shown at a higher magnification in the lower panel (**B**,**D**). These sections show relatively high (**B**) and low (**D**) root colonization. Visible idioblasts (I) containing dark phenolics. (**E**–**H**) Electron micrograph of an arbusculated cortex cell of a mycorrhizal Anthyllis root. (**E**,**G**) The graphs show sections of several arbuscule branches (arb) of functioning arbuscule, and degenerated arbuscule (darb), an arbuscule trunk (at) and intraradical hyphae (Ih) within intercellular spaces. (**F**,**H**) The graphs show thick-walled intercellular space with intraradical hyphae in M roots (**F**) and thin-walled cells in NM roots. En—endodermis, I—idioblast, RN—root nodule.

Approximately 20 fragments of Anthyllis roots with nodules collected from M and NM sites were analyzed by light (LM) and transmission electron microscopy (TEM). LM analysis showed that in both ecotypes of Anthyllis roots clearly defined nodules where formed, and opposite the nodules the root cortical cells were inhabited by AMF. The nodules were most likely inhabited by previously identified bacteria (*Rhizobium* and/or *Bradyrhizobium*) [29,38]. The ecotypes differed in the degree of root colonization by AM fungi, showing more intensive mycorrhization in the roots of metal-treated plants. Inside the roots of M plants, the AMF formed numerous intraradical hyphae, arbuscules and vesicles (Figure 2A,B). Moreover, M roots showed numerous phenol-containing idioblasts in the root cortex. NM roots showed relatively light root colonization (Figure 2C,D). TEM analysis revealed normal intraradical AMF structures inside M and NM roots; however, metal-treated roots showed thicker host cell walls compared to NM roots (Figure 2E–H).

### 2.4. Mycorrhizal Status, Spore Density, and Glomalin-Related Soil Protein Determination

**Figure 3:**
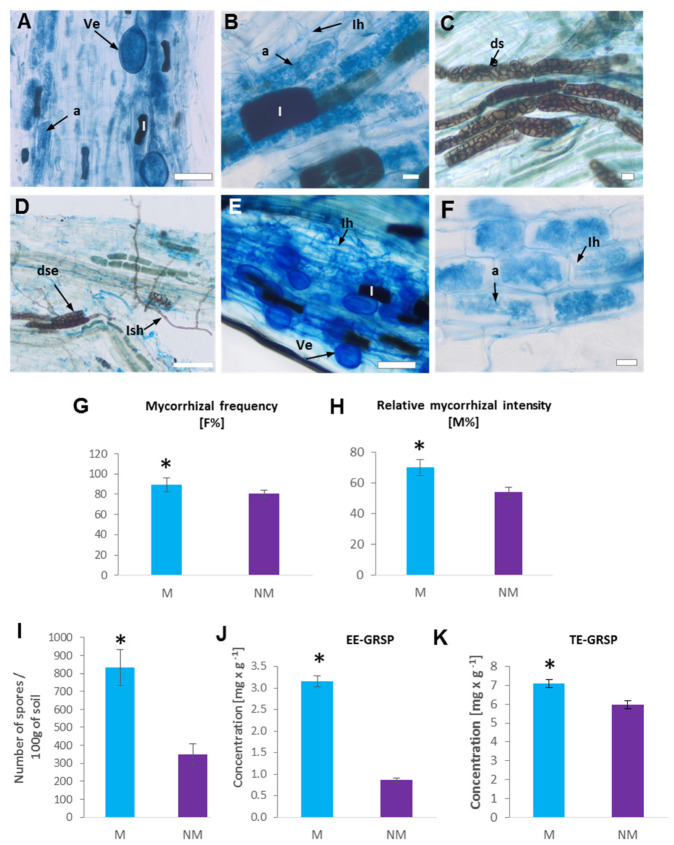
Mycorrhizal structures (**A**–**F**) and mycorrhizal parameters (**G**–**K**) in the roots and soil samples of calamine (M) (**A**–**D**) and non-polluted (NM) (**E**,**F**) Anthyllis ecotypes. (**A**–**D**) Aniline-blue stained roots of M plants colonized by arbuscular mycorrhizal fungi (**A**,**B**) and dark septate endophyte (DSE) fungi (**C**,**D**). Visible numerous idioblasts (I) with phenolics in cortex cells and fungal structures including vesicles (Ve), arbuscules (a) and intraradical hyphae (Ih). (**C**,**D**) Intracellular darkly pigmented septate hyphae (Ish) and microsclerotia (dse) of DSE fungi were occasionally detected. (**E**,**F**) Roots of NM plants colonized by AMF. Visible idioblasts (I) with phenolics and fungal structures including vesicles (Ve), arbuscules (a) and intraradical hyphae (Ih). Scale bar = 200 μm. (**G**,**H**) Mycorrhizal parameters of aniline blue stained roots of Anthyllis plants from Zn-Pb waste substratum (M) and reference non-polluted site (NM): G—mycorrhizal frequency (F%); H—relative mycorrhizal intensity (M%). (**I**) Total number of spores (in 100 g of soil), (**J**) easily extracted glomalin-related soil proteins (EE-GRSP), and (**K**) total extracted glomalin-related soil proteins (TE-GRSP) in metal-contaminated (M) and non-polluted (NM) soils. Data are presented as mean ± SE from *n* = 3; asterisk shows significant differences according to *t*-test (*p* < 0.05).

The evaluation of root colonization allowed the verification of the presence of typical for *Arum*-type AMF structures (arbuscules, hyphae and vesicles) inside the cortex roots of both Anthyllis ecotypes (Figure 3A–F). Both mycorrhizal parameters, mycorrhizal frequency F% and intensity of root cortex colonization M%, estimated on the basis of aniline blue staining, were significantly higher for the metal-treated roots than the control roots (Figure 3G,H).

Spore density in the rhizosphere varied among the soil samples, with 833 spores 100 g^−1^ soil for calamine substrate and 350 spores 100 g^−1^ soil for reference area. One way ANOVA showed a significant difference in spore density (*p* < 0.05) among the study sites. Thus, a significantly higher number of spores was observed in the rhizosphere of the M ecotype compared to the NM one (Figure 3I).

A significantly higher glomalin content was observed in the rhizosphere of M Anthyllis plants, compared to that of the non-polluted site (Figure 3J,K). The concentrations of easily extractable GRSP (EE-GRSP) and total GRSP (T-GRSP) in the rhizosphere soil of calamine-colonizing *A. vulneraria* were significantly higher than those of the non-polluted site. Our analysis reveals a statistically higher (about 7.10 mg g^−1^) concentration of T-GRSP in M soil than in NM soil, in which it reached 5.10 mg g^−1^. We also observed a statistically higher (3.2 mg g^−1^) concentration of EE-GRSP in M soil than in NM one, in which it reached 0.9 mg g^−1^ (Figure 3J,K).

### 2.5. Analysis of DGGE Profiles

**Figure 4:**
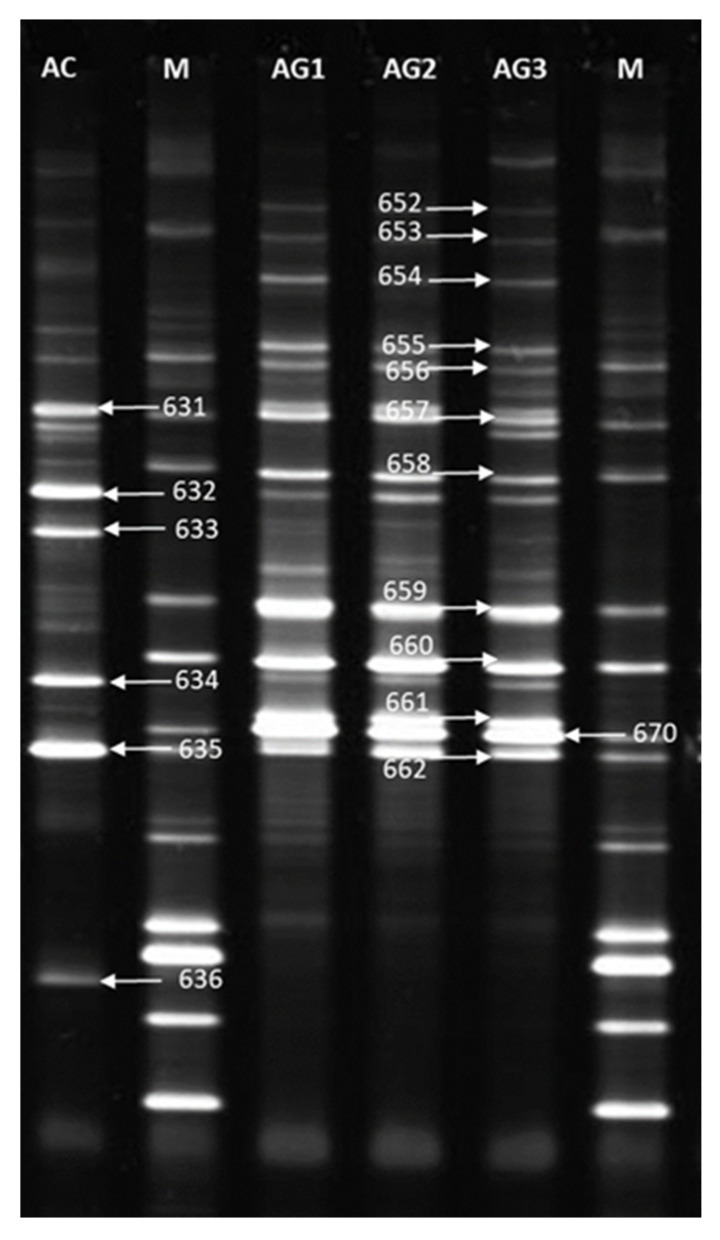
DGGE profile of 18S rDNA fragments from tested Anthyllis root samples. Lanes M: DGGE marker; lane AC: NM control; lanes AG1, AG2, AG3: M calamine heap, samples 1–3.

As a result of separation of PCR products in polyacrylamide gel, DNA profiles characterizing the presence of AM fungi in the roots of *A. vulneraria* plants were obtained (Figure 4). For the NM control sample 6 bands were obtained. For M root samples of plants from the calamine heap, 12 bands were obtained, with the DNA profiles for the three plants from the calamine heap being the same. The DNA profiles obtained for the control plant differed from the DNA profiles of the calamine plants.

### 2.6. Phylogenetic Analysis Based on Sequences Obtained from DGGE Bands

**Figure 5:**
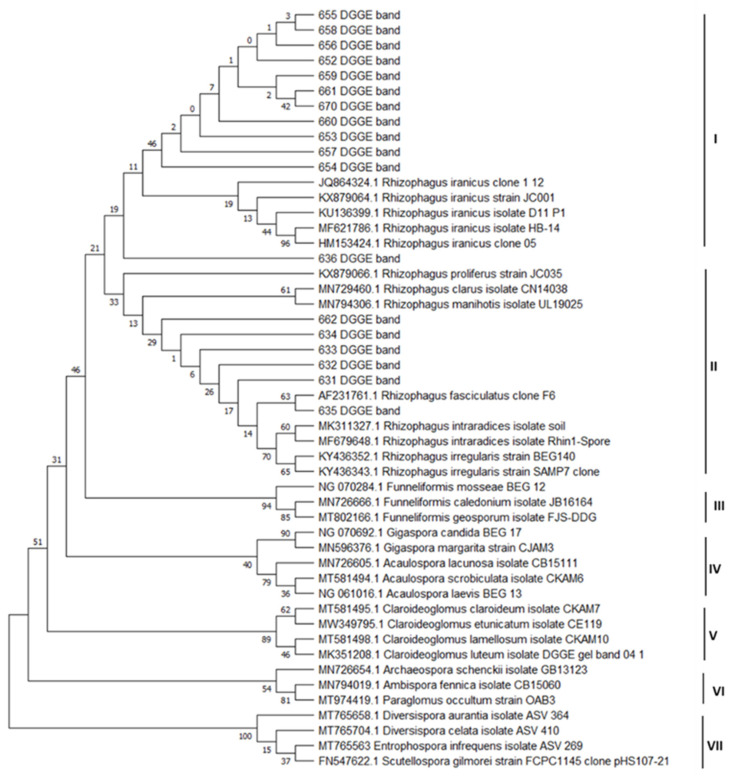
Phylogenetic tree of AMF partial SSU rRNA gene sequences showing the phylogenetic relationships between sequences obtained from DGGE bands and species of arbuscular mycorrhizal fungi. The numerical values (expressed as a percentage of 1000 repetitions) represent the percentage of generated dendrograms that had identical branching.

NS31-GC/Glo1 amplicons obtained from plant roots generated PCR-DGGE bands, which after excision from the gel, reamplification and sequencing yielded a total of 18 sequences. The dendrogram obtained from the sequences obtained in this study and the AMF sequences from the NCBI database enabled the identification of 7 clades (Figure 5). Sequences obtained in this study were grouped in clade I and clade II along with sequences of AMF species from the NCBI database. Five clades were created exclusively from the sequences of species from the NCBI database and belonging to the class Glomeromycetes.

In clade I, sequences obtained from roots of Anthyllis plants growing on a calamine heap (652–661 and 670 DGGE bands) clustered to sequences from the NCBI database, belonging to the species *Rhizophagus iranicus*. One sequence obtained from the roots of the control plant *A. vulneraria* (636 DGGE band) was also included in this clade.

In clade II, sequences from the control plant *A. vulne*raria (631–635 DGGE bands) clustered to species belonging to the genus *Rhizophagus* (*R. proliferus*, *R. clarus*, *R. manihotis*, *R. fasciculatus*), with one of these sequences (635 DGGE band) showing very strong association with the species *R. fasciculatus*. One sequence obtained from the roots of the *Anthyllis vulneraria* plant growing on a calamine heap (662 DGGE band) was also found to be related to clade II.

The remaining clades III–VII were formed from sequences of species of arbuscular mycorrhizal fungi of the genera *Funneliformis* (clade III), *Gigaspora*, *Acaulospora* (clade IV), *Claroideoglomus* (clade V), *Archaeospora*, *Paraglomus* (clade VI), *Diversispora*, *Entrophosphora* and *Scutellospora* (clade VII).

In the present work, DGGE profiles exhibited bands in all root samples, indicating that AMF successfuly colonized the roots of the plants growing on soils contaminated with heavy metals. In the roots of *A. vulneraria* plants, AM fungi belonging to the class Glomeromycetes, order *Glomerales*, family *Glomeraceae*, genus *Rhizophagus* were identified. AM fungi belonging to other orders of the Glomeromycetes class were not found. Phylogenetic analysis of the obtained sequences enabled the identification of similar fungi inhabiting M and NM Anthylis roots to the genus *Rhizophagus,* including the species *R. iranicus* and *R. fasciculatus* (Table 2).

## 3. Discussion

Mycorrhiza is an important factor enabling the adaptation of plants to sites contaminated with heavy metals [33]. The mycorrhization of legumes spontaneously colonizing metal-polluted areas is affected by environmental factors such as heavy metal concentration, drought, access to nutrients, soil pH, as well as specificity of the host [33]. The calamine ecotype of *A. vulneraria* is considered a metal-tolerant plant, which can tolerate very high amounts of toxic metals. In this study, AMF communities and their symbiotic interactions were analyzed in the roots of two Anthyllis ecotypes, plants growing on Zn-Pb-contaminated (M) calamine wastes and on a non-polluted reference site (NM). In our earlier study, we had proved that calamine wastes colonized by *A. vulneraria* are highly contaminated with Zn, Pb and Cd [5,29]. In the present study, soil chemical analysis confirmed that the tailings showed N and P deficiency, together with high concentrations of Ca and low concentrations of K and Mg (Table 1). Thus, the unfavourable conditions of this environment impose the need to compete for nutrients and other resources, which are evidently limited. Our study shows that, like other legume plants growing on metal-polluted tailings, Anthyllis plants form effective symbiotic associations with nitrogen-fixing rhizobia (with effective root nodules). Our earlier analyses of calamine Athyllis nodules had led to the discovery of naturally metal-resistant rhizobia, *Rhizobium* and *Bradyrhizobium* [29,38]. In this study Anthyllis plants grown on calamine tailings had in shoots a similar high N content as control plants (Table 1). Thus, biological nitrogen fixation (BNF) by rhizobia provides legumes with an additional source of nitrogen, especially on poor calamine tailings, but requires a high input of energy and phosphorus [47]. It has been shown that AM fungi stimulate BNF through improved phosphorus acquisition [47]. Our results confirm this statement. The P content together with K, Ca, and Mg in the shoots of calamine plants were significantly higher compare to control plants. These results suggest that the mycorrhizal root nodules of the calamine Anthyllis ecotype are occupied by adapted species of AM fungi effective in nutrients aquisition despite metal contamination. The amount of phosphorus supplied by different AMF species varies and this may be a key factor in the selection of a more efficient fungal partner for the legumes on metal-contaminated sites [13,48]. However, not much is known about AMF communities inhabiting the roots of legumes, and the mycorrhization status of legume plants colonizing metal-contaminated wastes has been poorly explored.

Because of the similar soil pH (7.1 and 7.5) [5] and low nitrogen and phosphorus content of the calamine tailings and the unpolluted reference area, the mycorrhizal colonization of roots of calamine-inhabiting plants and AMF sporulation seem to be correlated more with the heavy metal content than other soil parameters. The AMF colonization level of the roots growing in the calamine substrate was significantly higher than of the control roots (Figure 2). Additionally, we revealed that the amount of AMF spores in the rhizosphere of *A. vulneraria* in the tailings area was high (mean of 833 per 100 g of soil). Soil pH between 7.1 and 7.5 is suitable for spore germination of most of AM fungi species [49]. García-González et al. [50] have shown that physicochemical characteristics of soils can generate variations in the number of spores. High spore production may constitute an adaptation mechanism of AM fungi under stress conditions [51]. In the rhizosphere of M Anthyllis plants we also observed a higher glomalin content, both extractable and total one, compared to the non-polluted site (Figure 3). The observed increase in the amount of glomalin is related to the higher level of mycorrhization of calamine-colonizing plants. Glomalin is only detected in the roots infected by AM fungi. When the AMF presence in roots is low, the glomalin concentration decreases together with the concentration of AMF hyphae [52]. Many studies have shown the essential role of glomalin in soil aggregation and stabilisation of heavy metals such as Al, Cu, Pb or Cd in soil, thus improving plant growth on metal-polluted sites [52,53]. The higher glomalin content under metal stress observed in this study indicated that *AM fungi* were able to sequestrate bioavailable metals in the rhizosphere soil and thus reduced the migration of metals from the rhizosphere into the Anthyllis roots. The immobilization of metals in mycorrhizal roots was confirmed by dithizone staining (Figure 1). We showed that the nodules and root endodermis and intraradical fungal structures were the main sites of heavy metal accumulation (Figure 1). Similar sites of metal immobilization in intraradical fungal structures rather than in root cells of plants grown in metal-contaminated substrates have been observed [54,55]. We found the presence of metal ions around vesicles and arbuscules, and inside intracellular hyphae in the mycorrhizal roots of M plants. The AM fungal wall, with its major components, i.e., chitin and glomalin, serves as a deposition site for potentially toxic ions [56,57]. The fungal wall is responsible for 50% of the metal ions retained by AMF [57]. The mechanisms of metal tolerance in fungi also include intracellular sequestration by metallothioneins and phytochelatins [12,20,21,22,23]. Thus, by retaining metals in their hyphae, AM fungi are able to limit the transfer of metals to the shoots of the host plant [58]. This is in agreement with our previous studies. We had shown that the nodules in Anthyllis roots were the main sites of metal accumulation [5]. In metal-treated Anthyllis plants, some metals were also retained by the root endodermis, which is associated with the presence of the Casparian strip. Roots whose endodermis contains the Casparian strip limit the apoplastic metal ion transport to the shoots [47]. The TEM analysis carried out in this study showed thickening of the walls of cortex cells in the roots of metal-treated plants (Figure 2). Wall-thickening is a common defence strategy of legumes to cope with heavy metals [5,59]. It has been shown that AM fungi induce the biosynthesis of the cell wall in the roots of the host plant, which promotes metal reduction because of the increase in the thickness of the cell wall and the area for metal absorption [60]. This adaptation mechanism of calamine-colonizing Anthyllis plants based on wall thickening, which prevents the entry of toxic metals into the cells, together with AMF-induced metal immobilization, makes it possible for these plants to growth on metal-contaminated soils.

In the present study, despite the presence of AMF in M roots, the dark septate endophyte (DSE) fungi (hyphae and microsclerotia) were occasionally also detected (Figure 3). The DSEs occurred sporadically and were present mainly in those root fragments where AMF colonization was low. DSE fungi are often observed in the roots of plants inhabiting stressful environments [20]. DSEs are abundantly present in metal-polluted environments and some of them demonstrate high resistance to heavy metals [20]. Like AM fungi, DSEs facilitate the supply of nutrients to the host and enhance plant growth on metal-contaminated soils [17,18]. It is possible that DSE fungi can improve the growth of Anthyllis on calamine heaps, especially when AMF root colonization is poorly developed, but this requires a detailed study, and thus a thorough phylogenetic analysis of DSEs will follow in our further research.

We used the PCR-DGGE method to identify the observed AM fungi (Figure 4 and Figure 5). In our study, the set of primers used (NS31/Glo1) amplified a small ribosomal gene fragment. This set of primers amplifies all genera from the family *Glomeraceae*, except *Septoglomus*, and also genera from other AMF families such as *Acaulosporaceae, Ambisporaceae, Claroideoglomeraceae, Gigasporaceae, Paraglomeraceae* [45,61]. The PCR-DGGE, sequencing and BLAST analysis showed that the roots of both Anthyllis ecotypes were colonized with the same species of AM fungi belonging to the *Glomeraceae*. The *Glomeraceae* family is the largest AMF family and has been found to be adaptive to different environments [45]. The obtained results confirm that AM fungi from the *Glomeraceae* family can adapt to an environment heavily polluted with metals [39,54]. The phylogenetic analysis of the obtained sequences enabled the identification of similar fungi inhabiting the roots of *A. vulneraria* plants growing on the calamine heap and reference site to the genus *Rhizophagus,* including the species *Rhizophagus iranicus* and *R. fasciculatus* (Figure 4 and Figure 5, Table 2). Del Val et al. [62] had shown that metal tolerance can vary among the different ecotypes within the same AMF species. Pawloska et al. [39] identified in the calamine substrate *Glomus aggregatum, G. constrictum, G. fasciculatum, G. pansihalos*, and observed unidentified *Glomus* sp. and *Entrophospora* sp. In our erlier study, the fragment length analysis of the ITS1-5.8S-ITS2 rRNA gene region had revealed that both the rhizosphere and the roots of calamine-inhabiting *Trifolium repens* showed a high genetic diversity of mycorrhizal fungi [30]. A study of *Euphorbia cyparissias* growing on the Bolesław calamine substrate using polymerase chain reaction with specific primers had revealed the presence of *G. moseae, G. intraradices, G. claroideum, G. gerdemannii* and *Paraglomus occultum* [54]. Cornejo et al. [63] found that metal-tolerant AM fungi isolated from contaminated soils coped better with metal toxicity than those isolated from unpolluted soils. Thus, the high metal tolerance by these fungi may be a key factor in the selection of an efficient fungal partner for *A. vulneraria* plants on metal-contaminated sites.

## 4. Materials and Methods

### 4.1. Sampling and Soil Characteristics

Plants and soil samples of *A. vulneraria* were collected in July 2018 from two sites, a metal-polluted (referred to as M) calamine heap in Bolesław, southern Poland (50°17′ N 19°29′ E), and from an unpolluted reference site (referred to as NM)—a disused stone-pit in Kazimierz Dolny (51°19′ N 21°56′ E). Plants were sampled at the flowering stage and each plant was treated as a separate sample. The investigated, over 100-year-old, calamine tailings in Bolesław are composed mainly of the Triassic oolitic limestone and metalliferous dolomite (calamine) from the mined ore [46,64], and the NM stone-pit reference site also consists of limestone. Determination of mineral content was performed by an external company. The samples were sent to Bureau Veritas Mineral Laboratories (Canada) for plasma mass spectrometry (ICP-MS) analysis after mineralization of samples, according to our earlier protocol [5]. We found that M plants grew on tailings with pH 7.1–7.3, in the presence of toxic amounts of Zn (up to 42,983 mg·kg^−1^) and Pb (up to 2297.9 mg·kg^−1^), while on the NM reference site with pH 7.1–7.5 the metal content was very low: Zn (up to 55.6 mg·kg^−1^) and Pb (up to 10.4 mg·kg^−1^) [5,29]. On both sites the substrate was stony with a thin humus layer associated with densely vegetated turfs. The total N content in the shoots and soils was measured by the Kjeldahl method using a Vapodest analyzer, model VAP 30 (Gerhardt, Bonn, Germany).

### 4.2. Anatomical and Cytological Analyses

Fragments of *A. vulneraria* roots with nodules from the waste heap and the reference area were fixed, post-fixed in 1% OsO_4,_ dehydrated in increasing concentrations of ethanol, embedded in Epoxy resin and sectioned for light microscopy (LM, AX70 Provis, Olympus Poland) and transmission electron microscopy (TEM, Morgagni) according to our earlier protocol [5].

### 4.3. Histochemical Metal Ion Localization—Dithizone Staining

For histochemical heavy metal localization, randomly sampled roots with nodules were stained with dithizone (Sigma-Aldrich, St. Louis, MO, USA), which forms red complexes with metal (Pb, Cd) ions. Next, the roots where washed with deionized water and hand sections were observed under a LM (AX70 Provis, Olympus Poland, Warszawa, Poland) [5].

### 4.4. Spore Density

Spores from M and NM *A. vulneraria* rhizosphere were isolated using the wet sieving and decantation method [65,66,67]. Soil solutions were filtered through a column of sieves (0.5 mm, 0.125 mm, 0.063 mm and 0.045 mm), and the fractions of soil remaining on the successive sieves were washed away with distilled water into Petri dishes. The prepared samples were examined under a binocular stereomicroscope (NikonSMZ 800), and with the help of a micropipette the spores of mycorrhizal fungi were picked out and counted per 100 g of rhizosphere soil.

### 4.5. Glomalin Extraction

Total and easily extractable glomalin-related soil proteins (T- and EE-GRSP) were extracted from *A. vulneraria* rhizosphere samples according to the Jia et al. [68] method with some modifications. EE-GRSP was extracted from 1 g sieved soil sample with 8 mL of 20 mM of sodium citrate (pH 7.0) and then autoclaved for 30 min. (at 121 °C and 0.11 MPa) and centrifuged at 10,000× *g* for 5 min. The T-GRSP was obtained by repeated (3 to 5) extraction from 1 g of sieved soil sample with 8 mL of 50 mM sodium citrate, pH 8.0 at 121 °C for 60 min. After each autoclaving cycle, the supernatant was removed by centrifugation and stored. Extracts from each replicate were pooled and analyzed, and the protein in the supernatant was determined by the Bradford test with BSA (bovine serum albumin) as a standard.

### 4.6. Mycorrhizal Colonization

Roots were stained with 0.05% aniline blue in 80% lactic acid according to the Phillips and Hayman [69] method. For each sample, 30 stained root fragments approximately 1 cm long were randomly chosen, mounted on slides in glycerol:lactic acid (1:1) and pressed using cover slides. Mycorrhizal colonization in the roots and AM morphology were analyzed under a LM (Olympus, Provis) according to the Trouvelot et al. [70] method. Mycorrhizal frequency (F%, the frequency of mycorrhiza in all root segments) and mycorrhizal root cortex colonization intensity (M%, total number of root segments, in which the degree of colonization by mycorrhizal structures was 5) were calculated using the program MYCOCALC (http://www2.dijon.inra.fr/mychintec/Protocole/Workshop_Procedures.html, accessed on 1 January 2018).

The presence of fungal dark septate endophytes (DSE) was identified on the basis of dark pigmented sclerotia and septate hyphae [71].

### 4.7. DNA Extraction from Roots

DNA was isolated from 100 mg of each of the root samples using a Plant & Fungi DNA Purification Kit (EURx, Gdańsk, Poland). The DNA concentration was measured spectrophotometrically at 260 nm. For further analysis, samples with a DNA concentration of 10 ng ∙ μL^−1^ were prepared.

### 4.8. Nested PCR/PCR Conditions of DNA Amplification

PCR reactions were conducted with primers amplifying the 18S rRNA gene. DNA isolated from plant roots were first amplified with the AMF specific primer AM1 and the universal eukaryotic primer NS31 [72,73]. Reactions were performed in 30 thermal cycles (94 °C 30 s, 66 °C 1 min., 72 °C 1 min. 30 s). Amplification product from the first PCR reaction was diluted 1:10 and 1 µL of the dilution was used in a second round of PCR with the use of primers NS31-GC [73] and Glo1 [74]. Reactions of the second PCR were performed in 35 thermal cycles (94 °C 45 s, 52 °C 45 s, 72 °C 1 min.). The reactions were conducted in a total volume of 20 μL, containing 1× reaction buffer, 0.2 mM dNTPs, 0.2 μM of each primer, 0.5U of Taq DNA polymerase (AmpliTaq Gold^®^ DNA Polymerase, Applied Biosystems™, Waltham, MA, USA). The results of the reactions were checked on 1.2% agarose gel. The agarose gels were stained with ethidium bromide and visualized under UV light (GelDoc-It^®^Imaging System, UVP, Upland, Austin, TX, USA).

### 4.9. DGGE Analysis of AMF Communities

DGGE analysis was performed using the DCode™ Universal Mutation Detection System (Bio-Rad, Herkules, CA, USA). 15 µL of PCR products were used for the analysis. Reaction products were separated in polyacrylamide gels (37.5:1 acrylamide:bisacrylamide) at a concentration of 8% and 35–55% chemical gradient. Electrophoresis in 1xTAE buffer was conducted for 990 min. (16.5 h) and a voltage of 50 V. The polyacrylamide gels were stained with SYBR GREEN I nucleic acid gel stain (Sigma-Aldrich) (1: 10,000 dilution) for 30 min. and photographed using GelDoc-It^®^Imaging System, UVP, Upland, Austin, TX, USA) [75].

### 4.10. Sequencing of 18S rDNA Fragments (of DGGE Bands)

The bands selected for reamplification and sequencing were common bands, the most intense and unique. The bands excised from the gel were suspended in 50 µL sterile, deionised water. PCR reactions were performed with the NS31/Glo1 primers using 2 μL of the suspension as a template. The results of the reactions were checked on 1.2% agarose gel. The reaction products were turned over to a standard Sanger (Genomed S.A., Warsaw, Poland) sequencing technique. The quality of the obtained sequences was evaluated using the BioEdit program v7.2.5 (Ibis Biosciences).

### 4.11. Phylogenetic Analysis

Reference sequences of 26 AMF species were selected for phylogenetic analysis. Multiple 18S rDNA gene sequence alignments were performed with MEGA X using CLUSTAL W. A phylogenetic tree was constructed using the neighbour-joining method (analysis model: Tamura-Nei with Gamma distribution) with a bootstrap analysis based on 1000 resampling of the dataset.

### 4.12. Statistical Analysis

Data were statistically evaluated using STATGRAPHICS Plus 5.1. Significant differences among treatments were tested with Tukey’s (HSD) test and analysis of variance (ANOVA).

## 5. Conclusions

The results of this work indicate that the mycorrhizal root nodules of the calamine Anthyllis ecotype are occupied by adapted species of AM fungi effective in nutrients aquisition despite metal contamination. These special calamine AMF ecotypes play an important role in the adaptation of *Anthyllis vulneraria* to the extreme conditions in calamine tailings. The knowledge of the AMF associated with legumes on metal-polluted sites will contribute to the selection of unique endosymbionts to help formulate remediation strategies for metal-contaminated soils.

## Figures and Tables

**Table 1:** Chemical properties of *Anthylis vulneraria* shoots and rhizosphere soils sampled from metal-polluted calamine tailing (M) and non-polluted (NM) reference site.

Concentrations	Calamine Tailing (M)	Un-Polluted Soil (NM)	Shoots (M)	Shoots (NM)
N g kg^−1^	1.01 ± 0.09 a	0.99 ± 0.08 a	11.8 ± 0.52 a	11.6 ± 0.25 a
P %	0.03 ± 0.01 b	0.02 ± 0.01 b	0.14 ± 0.05 a	0.09 ± 0.01 b
K %	0.10 ± 0.01 a	0.03 ± 0.03 b	1.85 ± 0.15 a	1.15 ± 0.12 b
Ca %	5.6 ± 0.24 b	5.9 ± 0.51 b	3.89 ± 0.52 a	2.21 ± 0.42 b
Mg %	2.9 ± 0.14 a	0.44 ± 0.01 b	1.46 ± 0.16 a	0.21 ± 0.05 b

Values are presented as means ± SD. Soil was collected from the top 20 cm layer. Different letters indicate significantly different means at *p* < 0.05 according to one-way ANOVA and post-hoc Tukey’s test.

**Table 2:** Identification of AMF fungi based on phylogenetic analysis of sequences obtained from DGGE bands in root samples of *Anthyllis vulneraria* growing on the calamine heap (M) or on the non-polluted site (NM).

Sample	DGGE Bands
Anthyllis NM (AC)	631—*Rhizophagus* sp.; 632—*Rhizophagus* sp.; 633—*Rhizophagus* sp.; 634—*Rhizophagus* sp.; 635—*Rhizophagus fasciculatus*; 636—*Rhizophagus iranicus*
Anthyllis M (AG1)	652—*Rhizophagus iranicus*, 653—*Rhizophagus iranicus*, 654—*Rhizophagus iranicus*, 655—*Rhizophagus iranicus*, 656—*Rhizophagus iranicus*, 657—*Rhizophagus iranicus*, 658—*Rhizophagus iranicus*, 659—*Rhizophagus iranicus*, 660—*Rhizophagus iranicus*, 661—*Rhizophagus iranicus*, 662—*Rhizophagus* sp., 670—*Rhizophagus iranicus*
Anthyllis M (AG2)	652—*Rhizophagus iranicus*, 653—*Rhizophagus iranicus*, 654—*Rhizophagus iranicus*, 655—*Rhizophagus iranicus*, 656—*Rhizophagus iranicus*, 657—*Rhizophagus iranicus*, 658—*Rhizophagus iranicus*, 659—*Rhizophagus iranicus*, 660—*Rhizophagus iranicus*, 661—*Rhizophagus iranicus*, 662—*R. fasciculatus*, 670—*Rhizophagus iranicus*
Anthyllis M (AG3)	652—*Rhizophagus iranicus*, 653—*Rhizophagus iranicus*, 654—*Rhizophagus iranicus*, 655—*Rhizophagus iranicus*, 656—*Rhizophagus iranicus*, 657—*Rhizophagus iranicus*, 658—*Rhizophagus iranicus*, 659—*Rhizophagus iranicus*, 660—*Rhizophagus iranicus*, 661—*Rhizophagus iranicus*, 662—*R. fasciculatus*, 670—*Rhizophagus iranicus*

## Data Availability

The data presented in this study are available on request from the corresponding author.

## Abbreviations

AMF	arbuscular mycorrhizal fungi
DSE	dark septate endophyte fungi
M	metallicolous ecotype
NM	non-metallicolous ecotype
TEM	transmission electron microscope
